# A Medical Journal for the World's Health Priorities

**DOI:** 10.1371/journal.pmed.1000072

**Published:** 2009-04-28

**Authors:** The PLoS Medicine Editors

## Abstract

The *PLoS Medicine* editors lay out their renewed vision for the journal–of taking an evidence-based approach to publishing on topics of global health priority, which encompass the social, environmental, and political determinants of health, as well as the biological.

In the age of the Internet, five years can seem like an eternity. *PLoS Medicine* issued its first call for papers five years ago and the inaugural issue went live online five years ago this October—for those of you who are nostalgic, check out the original call for papers [1]. Anniversaries often prompt reflection, and over the past few months we've taken a close look at our original plans for *PLoS Medicine*, what's happened since the journal launched, and most importantly how the journal should evolve in the future. We now propose a refocusing of the journal's priorities that will, we believe, align them more closely with the world's health priorities.

To recap, we had two main aims when we launched *PLoS Medicine*. First, to provide an open-access alternative to top-tier subscription medical journals such as the *New England Journal of Medicine* and the *Lancet*, in the same way that *PLoS Biology* was launched a year earlier to challenge the dominance of general subscription science journals such as *Science* and *Nature*. There was a niche in the market that desperately needed filling—a need to prove that the new model of open-access publishing was compatible with the highest standards of medical journal publishing. In our inaugural editorial, “Prescription for a Healthy Journal” [2], we acknowledged that the Internet was not only the technology driving *PLoS Medicine*'s inception but also was central to its ethos by enabling “the revolutionary idea of anyone being able to read any article.” Second, we had a vision for a journal that had a clear “priority of publishing papers on diseases that take the greatest toll on health globally.”

Has *PLoS Medicine* measured up to these aims and are these aims still valid? For the first, in the five years since *PLoS Medicine* launched, open-access publishing has matured from a seemingly unrealistic dream into a mainstream concept. We would argue that our presence has led other medical journals to begin to consider making some of their content free access (i.e., articles are free to read, but published under traditional restrictive copyright) or truly open access (free to read and published under a progressive copyright license allowing reuse) [3]. By 2005, a study published in *BMJ* [4] found that much of the literature was already freely available online. In 2008, John Willinsky argued further that “about twenty percent of the research literature published today ends up open access” [5] (though this may not be true open access). The Directory of Open Access Journals (http://www.doaj.org/) now contains 3,999 journals. When we launched, few authors were convinced of the merits of open-access or even free-access publishing compared with traditional publishing, and they rarely made publishing decisions based on access to their work. Now, however, authors are starting to make decisions about publication based on how accessible the work will be, and funders, governments, and institutions are routinely mandating open, or more frequently free, access to the research they support (see Peter Suber's blog Open Access News for an archive of relevant developments [http://www.earlham.edu/~peters/fos/fosblog.html]).

In addition to advancing open access, PLoS is building on the potential of the Internet to enable and enrich human interaction around scientific publications. A first step in this direction is the integration of all the PLoS journals on one publishing platform—as of last month *PLoS Medicine* is hosted on our in-house open-access platform, Topaz, which optimizes user interactions. We invite you to explore all the functionalities of Topaz, including the ability to comment on and rate the articles that we publish.

Within such a framework of access and technology, what should *PLoS Medicine*'s role be in the future, as the leading, fully open-access general medical journal? We have never found it sufficient just to fulfil our first aim as an open-access alternative to the other top medical journals—we have tried to distinguish *PLoS Medicine* from other journals in more active ways. One example: we stated at the outset that “we have decided not to be part of the cycle of dependency that has formed between journals and the pharmaceutical industry” [2]. We have broken that dependency by banning advertisements for drugs or medical devices; in addition, because our open-access license allows anyone worldwide to make unlimited copies of any paper, we cannot benefit from an exclusive reprint trade to drug companies (a clinical trial published in a subscription-based journal can earn that journal thousands if not hundreds of thousands of dollars in reprint sales).

To return to our second stated aim at the outset, we also intended that *PLoS Medicine* should be distinguished from other Western medical journals by its content. We always aimed to publish papers highlighting diseases that affect a large portion of the world's population: the “global burden of disease.” We remain guided by the conviction that research reports, especially those on work that most affects human health globally, must be available to all, and not restricted by access fees and legal barriers to reuse. We believe that the first five years of *PLoS Medicine* have brought substantial progress toward this goal, and we now want to focus our efforts on accomplishing it. Thus, from April 2009 we will take an evidence-based approach to identifying topics of global health priority, and will give highest priority to publishing studies that advance human health in these areas.

We will be guided in our editorial decision-making by sources providing evidence on the specific diseases and risk factors that cause the greatest burden worldwide. Such lists are of course not perfect, so we will not apply them rigidly, but will instead use them as a guide in prioritizing papers for publication (for examples, see [6]–[8]). Within these priority areas we will make decisions based on: (1) whether papers are likely to directly and substantially affect clinical practice or public health policy, or (2) whether they have profound implications for the direction of future, clinically relevant research. However, papers that report, for example, biochemical pathways or genetic associations without clear application to human health, would not reflect the new priorities of *PLoS Medicine*. We believe there are other open-access alternatives for such papers (including other PLoS journals [9]).

In taking such an approach we emphasise the need to look beyond just the biological causes of disease. Despite the stunning advances that have been made in understanding disease pathophysiology, improving diagnosis, and developing new therapies, human health remains inextricably intertwined with the environment—in its widest sense—in which we live. The conditions and risk factors that cause the highest burden of disease clearly reflect these interrelationships. For example, it would be simplistic to look only for the biological causes of the morbidity and mortality caused by perinatal conditions, diarrhoeal diseases, poor nutrition, or smoking. As the world faces up to the challenges of a changing climate, a turbulent economic system, and continued global conflict, we now wish to reinforce the important place in health research of work that encompasses the social, environmental, and political determinants of health, as well as the biological.

Some things will not change: for example, we will continue to prioritize papers that cover important topics in the ethics and reporting of research. And, as always, we will publish papers across the methodological spectrum, from observational research to basic pathophysiology. Further details of our editorial mission can be found on our Web site [10]. We welcome your feedback on this initiative. To give you a sense of our new scope, take a look at the papers we are publishing this week [11]–[16].

We believe our new, evidence-based approach will not only ensure that open-access publishing reflects the health priorities of the 21st century, but will also reaffirm and revitalize the long tradition of medical journals leading, rather than following, the debate over research priorities.
